# Conformational fitting of a flexible oligomeric substrate does not explain the enzymatic PET degradation

**DOI:** 10.1038/s41467-019-13492-9

**Published:** 2019-12-06

**Authors:** Ren Wei, Chen Song, Daniel Gräsing, Tobias Schneider, Pavlo Bielytskyi, Dominique Böttcher, Jörg Matysik, Uwe T. Bornscheuer, Wolfgang Zimmermann

**Affiliations:** 1 Department of Microbiology and Bioprocess Technology, Institute of Biochemistry, Leipzig University, Johannisallee 21-23, D-04103 Leipzig, Germany; 2 Department of Biotechnology and Enzyme Catalysis, Institute of Biochemistry, University of Greifswald, Felix-Hausdorff-Str. 4, D-17487 Greifswald, Germany; 3 Institute of Analytical Chemistry, Leipzig University, Linnéstrasse 3, D-04103 Leipzig, Germany

**Keywords:** Biocatalysis, Enzyme mechanisms, Enzymes, Molecular modelling, Biocatalysis

**Arising from** Joo et al. *Nature Communications* 10.1038/s41467-018-02881-1

Joo et al.^1^ have recently reported a crystal structure of a polyethylene terephthalate (PET) hydrolyzing enzyme (*Is*PETase) from *Ideonella sakaiensis* which has been described able to metabolize PET at 30 °C^2^. They proposed a PET degradation mechanism solely based on covalent computational docking of an oligomeric substrate—2-hydroxyethyl-(monohydroxyethyl terephthalate)_4_—(2-HE(MHET)_4_) into the substrate binding cleft of *Is*PETase without considering the motions and conformations of the PET polymer chain. Here we present a solid-state nuclear magnetic resonance (NMR) analysis of amorphous PET at the degradation temperature used by Joo et al.^1^, indicating that the highly stiff polymer chain can hardly resemble the suggested docking conformation of 2-HE(MHET)_4_. In correlation with the PET degradation performance obtained at the same temperature, *Is*PETase is unlikely to follow the catalytic mechanism proposed by Joo et al.^1^, which requires simultaneous binding and interaction of all four MHET substrate moieties with the binding site of the enzyme.

While *Is*PETase can hydrolyze amorphous PET, it showed almost no activity against the crystalline PET polymer^2^. The OC–CO torsion angle *Ψ* in the ethylene glycol (EG) units of amorphous and crystalline PET reveals distinct probability distributions^3^. According to the 2-HE(MHET)_4_ docking conformation described by Joo et al.^1^, a *trans* (*t*, *Ψ*_*t*_ ≈ 180°) content higher than 25% was obtained, considerably higher than the literature value of 14 ± 5% obtained for amorphous PET at ambient temperature^3^. In our study here, we determined the probability distribution of the OC–CO torsion angle *Ψ* in a commercially available amorphous PET material and obtained a *trans* to *gauche* (*Ψ*_*g*_ ≈ ±70°) ratio of 9:91 at 30 °C (Fig. 1a), the temperature at which Joo et al.^1^ performed their PET degradation experiments^1^. This value is in good agreement with the literature^3^ but significantly lower than the *t*/*g* ratio obtained in the 2-HE(MHET)_4_ docking conformation, suggesting that the latter conformation is rarely present in amorphous PET polymer chains. As a consequence, even if the target ester bond in 2-HE(MHET)_4_ between subsite I and subsite IIa can be correctly accessed by the catalytic triad of the *Is*PETase (Fig. 2b in Joo et al.^1^), residues in distal subsites IIb and IIc are unlikely to interact with the other two MHET moieties due to a more biased presence of *gauche* contents in the PET polymer. In a later publication dealing with the *Is*PETase structure^4^, Austin et al.^4^ have also shown induced fit docking conformations for a PET tetramer, which is equivalent to 2-HE(MHET)_4_ used by Joo et al.^1^. For the wildtype *Is*PETase and a double mutant (S238F/W159H) with increased PET hydrolyzing activity, different energetically most-favored conformations of the PET tetramer were demonstrated by Austin et al.^4^: a near-*trans* conformer for the EG unit directly flanking the target ester bond in the wildtype enzyme and a *gauche* conformer in the double mutant. Nevertheless, in contrast to Joo et al.^1^, Austin et al.^4^ did not report on the questionable interactions of distal PET repeating units with surrounding residues in their hypothesis of substrate binding and catalysis.

**Fig. 1:**
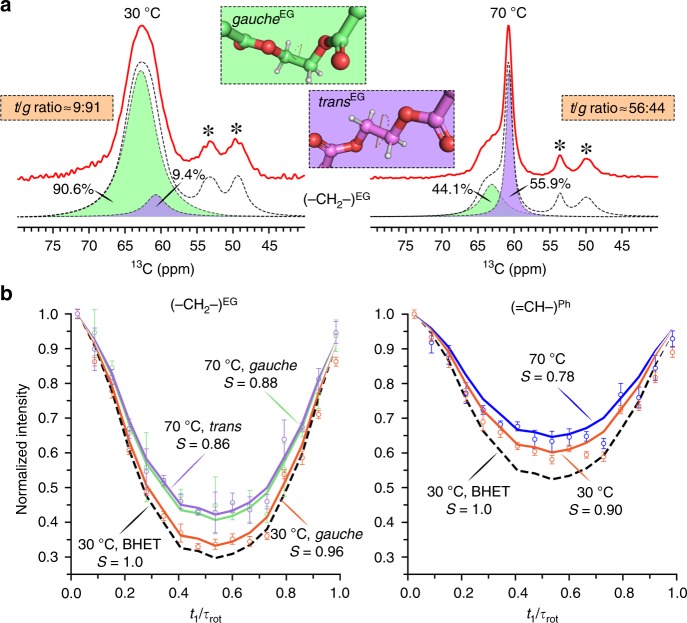
MAS NMR of amorphous PET powder at 30 °C and 70 °C. **a** ^13^C spectra in the range of 40–80 ppm showing the characteristic spectra for the EG carbons. The *trans* and *gauche* contents were quantified by fitting a Voigt function to the experimental spectra (red). The sum of the Voigt fits (shaded in purple and green, respectively, for *trans* and *gauche* conformers) is shown as dotted curve. The inset shows the EG fragment in the *trans* and *gauche* conformations. MAS sidebands are denoted by asterisks. **b** ^1^H–^13^C DIPSHIFT curves for phenylene and EG carbons acquired at a MAS rate of 8 kHz. The solid lines are the best-fit simulations and dashed lines represent the rigid limit. Experimental error bars were determined from the noise level. Order parameters *S* from the measured dipolar couplings are given.

**Fig. 2:**
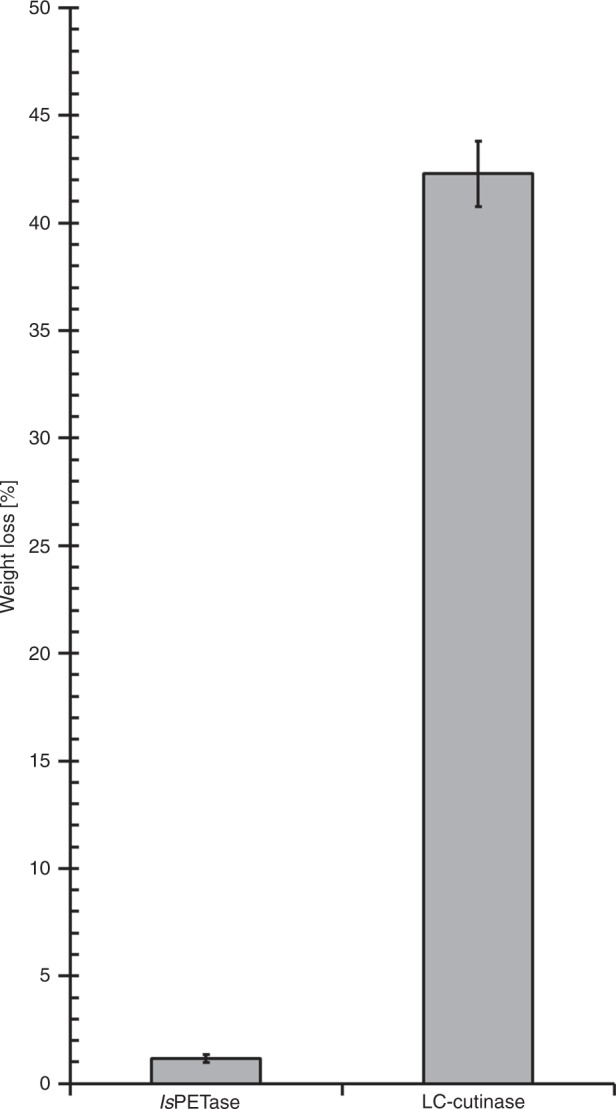
Weight loss of an amorphous PET film (~45 mg) as a result of enzymatic hydrolysis. Incubation with *Is*PETase and LC-cutinase was carried out under agitation for 24 h at 30 °C and 70 °C, respectively, i.e., at their optimal reaction conditions for PET degradation. Error bars indicate the standard deviation of triplet measurements.

Moreover, we examined the localized and large-scale cooperative main-chain motions in amorphous PET by magic-angle-spinning (MAS) NMR methods. The ^1^H–^13^C dipolar coupling order parameters *S* measured using 2D dipolar chemical shift correlation (DIPSHIFT) experiments were used to quantify the site-specific motions on a sub-microsecond timescale to describe the relative rigidity in a value range of 0 to 1^5^. Experimental data obtained with crystalline bis(2-hydroxyethyl) terephthalate (BHET) were used to define the rigid limit corresponding to *S* = 1. At 30 °C, the dominant *gauche* conformer in EG units of amorphous PET showed an order parameter of *S*_CH_ = 0.96, indicating that the transition from *gauche* to *trans* conformation was highly restricted (Fig. 1b). Therefore, conformational changes in PET segments in order to perfectly fit the substrate binding cleft of *Is*PETase as described by Joo et al.^1^ cannot occur at 30 °C. Nevertheless, we determined a more than 1% weight loss of the same amorphous PET film as a result of *Is*PETase-catalyzed hydrolysis following 24 h of incubation at 30 °C (Fig. 2). Based on the molecular dynamics simulations shown in another study^6^, amino acid residues and backbones involved in the substrate binding cleft of *Is*PETase are not allowed to move freely to fit any arbitrary rigid conformation of a PET polymer segment. Thus, *Is*PETase is likely to follow other binding and degradation mechanisms with regard to the PET hydrolysis at 30 °C than those proposed by Joo et al.^1^.

PET has a glass transition temperature above 70 °C^7^ which has been suggested as a more favorable reaction condition for enzymatic PET hydrolysis^8,9^. Yoshida et al.^2^ verified this by demonstrating a more than 100-fold higher release of UV-absorbing degradation products using LC-cutinase, a thermophilic homologous counterpart of *Is*PETase, against an amorphous PET sample than using *Is*PETase after an incubation time of 1 h at their individual optimal reaction conditions^2^. Similarly, we compared the degradation performance of both enzymes against the PET material used in this study and showed a more than 40-fold higher weight loss obtained by LC-cutinase after an incubation time of 24 h (Fig. 2). These differences in their PET degradation performance were considered to be strongly dependent on the polymer chain mobility and accessibility at different temperatures, as suggested previously also in another publication^8^. As shown in Fig. 1, we observed slightly larger amplitudes of motions in EG units at 70 °C indicated by the lower order parameters *S*_CH_ of 0.86 (*trans*) and 0.88 (*gauche*). As a consequence, the transition between *trans* and *gauche* conformers was allowed resulting in a *t*/*g* ratio of 56:44, a significantly higher value than that obtained at 30 °C. This indicated a transition tendency of PET polymers from a less-ordered state to a more-ordered one as a result of physical aging during the incubation at 70 °C, which is consistent with our recent publication^10^. Interestingly, the aromatic phenylene rings in amorphous PET exhibited lower order parameters with *S*_CH_ = 0.90 and 0.78 at 30 °C and 70 °C, respectively, indicating that the phenylene units in PET are more prone to motions compared with the flanking rigid EG units at both temperatures. This is in agreement with a previous publication reporting a phenylene ring flip motion in amorphous PET on a millisecond-to-second timescale^11^. Therefore, we hypothesize that the weak interactions between the aromatic phenylene units and the surrounding hydrophobic amino acid residues are more likely to facilitate the substrate binding requested for the subsequent enzymatic hydrolysis of PET rather than the perfect accommodation of a certain conformation of a polymer segment, especially at an ambient temperature around 30 °C.

## Methods

### Solid-state NMR analysis of amorphous PET powder

Amorphous PET films (product no. ES301445) were purchased from Goodfellow Cambridge Ltd. (Huntingdon, UK) and ground in a cryomill in the presence of liquid nitrogen. Particles with diameters of less than 0.25 mm were obtained by sieving and then used in the solid-state NMR analysis. All CP/MAS (cross-polarization magic-angle spinning) NMR experiments were performed at 9.4 T with a Bruker AVANCE III NMR spectrometer equipped with a 4-mm double-resonance MAS probe (Rheinstetten, Germany). A MAS rate of 8 kHz was maintained throughout the experiments. The ^207^Pb NMR resonance of lead nitrate was used as the thermometer to calibrate the temperature over the sample volume of the in-situ MAS probe at 8 kHz^12^. The MAS data were acquired respectively at 30 and 70 °C by using two individual samples. Each fine-powdered PET sample (~30 mg) was loaded into a 4 mm ZrO_2_ MAS rotor with Vespel cap and incubated at their individual target temperatures in the magnet for 24 h prior to the data acquisition. Optimized ^1^H and ^13^C 90° pulse lengths were 2.5 and 3.0 μs, respectively. The CP/MAS spectra were recorded with 2512 scans and a relaxation delay of 4 s, with optimized spin-lock pulses to satisfy both Hartmann–Hahn (HH) *n* = ±1 matching conditions, with 66 kHz effective ^13^C radio frequency (r.f.) lock field and 100–70% ramp on the ^1^H channel. Decomposition of methylene carbon resonances from the EG units provided direct quantification for the *t*/*g* ratio. The contact time was 2 ms. ^1^H–^13^C order parameters were measured using the 2D DIPSHIFT experiments^5^. The experiments were conducted with 256 scans and a recycle delay of 4 s. ^1^H–^1^H homonuclear dipolar decoupling was accomplished with phase-modulated Lee–Goldburg (PMLG) approach^13^. The PMLG block consists of 10 pulses with the following phases: 339.22°, 297.65°, 256.08°, 214.51°, 172.94°, 352.94°, 34.51°, 76.08°, 117.65°, and 159.22° (m5m shape in TopSpin library, Bruker). The PMLG5-optimized pulse was 2.07 μs and the r.f. decoupling field was set to 80 kHz. The PMLG scaling factor of 0.5 was determined based on adamantane *J*-splitting. For all experiments, swept-frequency two-pulse phase-modulation (SW_f_-TPPM) heteronuclear decoupling^14^ with a r.f. field of 100 kHz was used during the acquisition. ^13^C chemical shifts were externally referenced to the C(=O)O^−^ signal of solid tyrosine·HCl at 172.1 ppm. The DIPSHIFT dephasing curves were simulated using the SIMPSON program^15^. The rigid-limit values for both CH and CH_2_ spin systems were obtained by fitting the experimental curves of crystalline BHET in a site-specific manner. Spectral fitting was conducted with the MestReNova 12.0.0 program (Mestrelab Research, Santiago de Compostela, Spain).

### Enzymatic hydrolysis of amorphous PET films

A codon-optimized synthetic gene encoding *Is*PETase lacking the N-terminal signal sequence containing 27 residues was ordered from Genscript (Piscataway, USA) and subcloned into the pET-21b vector (Novagen, San Diego, USA). The recombinant *Is*PETase containing a C-terminal His_6_-tag was expressed in *Escherichia coli* Shuffle T7 Express (New England Biolabs GmbH, Frankfurt am Main, Germany). Briefly, *E. coli* cells were grown at 30 °C to an optical density (OD_600_) of 1, followed by induction in the presence of 0.1 mM IPTG at 16 °C for more than 12 h, and then purified by immobilized metal ion chromatography (IMAC) using TALON metal affinity resin (Takara Bio Europe, Saint-Germain-en-Laye, France) to homogeneity. LC-cutinase was recombinantly expressed in *E. coli* BL21(DE3) and purified by IMAC to homogeneity using Ni-NTA (Qiagen, Hilden, Germany) as previously described^16^. 5 μg of *Is*PETase and 50 µg of LC-cutinase were used to degrade a piece of amorphous PET film (Goodfellow Cambridge Ltd., 3 × 0.5 cm^2^, ~45 mg) using the optimal degradation conditions for the respective enzymes^2,16^. Briefly, *Is*PETase required 50 mM Na_2_HPO_4_-HCl at pH = 7 while LC-cutinase required 1 M K_2_HPO_4_/KH_2_PO_4_ at pH = 8. Degradation was performed by shaking the reaction vials on a thermoshaker TS1 (Biometra, Göttingen, Germany) at 1000 rpm for 24 h at 30 °C for *Is*PETase and at 70 °C for LC-cutinase. The reaction was stopped by cooling the samples on ice. PET films were washed sequentially with 0.1% aqueous SDS, ethanol and ultrapure water and then dried at 50 °C for 48 h before subjected to gravimetric weight loss determination. Degradation experiments using higher *Is*PETase concentrations up to 50 µg enzyme and lower LC-cutinase amount down to 5 µg enzyme were also prepared as control samples which led to significantly lower weight losses of the PET films (data not shown) than using the enzyme amounts mentioned above in main text.

## Data Availability

The datasets generated during and/or analyzed during the current study are available from the corresponding author on reasonable request.
